# The Great Recession and Children’s Mental Health in Australia

**DOI:** 10.3390/ijerph16040537

**Published:** 2019-02-13

**Authors:** Melisa Bubonya, Deborah A. Cobb-Clark, Daniel Christensen, Sarah E. Johnson, Stephen R. Zubrick

**Affiliations:** 1 School of Economics, Level 5, Social Sciences Building, University of Sydney, NSW 2006, Australia; deborah.cobb-clark@sydney.edu.au; 2 ARC Centre of Excellence for Families and Children over the Life Course, University of Queensland, Level 2, Cycad Building (1018), 80 Meiers Rd, Indooroophilly, QLD 4068, Australia; daniel.christensen@telethonkids.org.au (D.C.); stephen.zubrick@telethonkids.org.au (S.R.Z.); 3 Institute for the Study of Labor (IZA), Schaumburg-Lippe-Straße 5-9, 53113 Bonn, Germany; 4 Telethon Kids Institute, Northern Entrance, Perth Children’s Hospital, 15 Hospital Ave, Nedlands, WA 6009, Australia; sarah.johnson@telethonkids.org.au; 5 Centre for Child Health Research, University of Western Australia, Nedlands, WA 6009, Australia

**Keywords:** mental health, children, economic recession, macroeconomic, consumer sentiment, Australia, longitudinal studies

## Abstract

This paper analyzes the effects of “shocks” to community-level unemployment expectations, induced by the onset of the Great Recession, on children’s mental well-being. The Australian experience of the Great Recession represents a unique case study as despite little change in actual unemployment rates, levels of economic uncertainty grew. This affords us the ability to examine the effects of shocks to economic expectations independent of any actual changes to economic conditions. We draw on and link data from multiple sources, including several waves of the Longitudinal Study of Australian Children (2004–2010), a consumer sentiment survey, and data on local economic conditions. Using our purpose-built data set, we estimate difference-in-differences models to identify plausibly causal effects. We find, for boys, there is no detectable effect of community-level unemployment expectations shocks on mental health. For girls, however, there are modest increases in mental health problems and externalizing behaviors, as measured by the Strengths and Difficulties Questionnaire (SDQ). We additionally find no discernible change in mother’s psychological distress as a result of expectations shocks. These results are stable after controlling for actual labor market conditions.

## 1. Introduction

There is an extensive literature examining the effects of economic conditions on population health. In seminal work, Ruhm [1] found economic downturns in the United States to be associated with reduced mortality due in large part to a reduction in preventable deaths. Subsequent researchers also found mortality to be procyclical in other countries, though the findings from more recent studies are much less conclusive [2,3]. In contrast, there is little doubt that adult mental health worsens as macroeconomic conditions decline [4,5,6,7,8,9,10,11,12]. Researchers typically parameterize economic downturns using changes in labor market conditions (e.g., unemployment rates, mass layoffs), however, the relationship between macroeconomic conditions and population health holds more generally, including when the focus is on macroeconomic factors such as GDP and home foreclosure. Foreclosures, for example, have also been linked to hospital and emergency room visits [13]. Economic crises can be associated with chronic stress for many people which can produce both adaptive and dysfunctional psychological responses, such as depression [14]. Greater vulnerability to the mental health effects of economic crises exists among those with previous mental illness, the unemployed, migrants, ethnic minorities, children, young people, and the elderly [9,11,15].

Unfortunately, there is little consensus on the relationship between macroeconomic conditions and children’s health [16,17]. Increased unemployment is associated with more child abuse and neglect [18], an increased incidence of injuries [17], and worse child and adolescent mental health [16,17], but also lower obesity rates, especially for young children and adolescents [2]. Similarly, US studies examining the effect of aggregate unemployment rates versus anticipated job losses on infant health arrive at opposite conclusions. Babies conceived during periods of high unemployment rates appear to have better health outcomes (specifically, a reduced incidence of low and very low birth weight, fewer congenital malformations, lower postneonatal mortality) [19,20], yet the announcement of impending job losses appears to lead to a temporary decline in birth weight [21]. In California, the perception of job loss (that was not actually realized) was linked to increased rates of low birthweight [22]. 

This evidence that anticipated macroeconomic outcomes affect child health points to an important mechanism through which economic downturns may affect population health—economic fear and stress. Even if households do not personally experience job loss, they may experience an increase in job insecurity and stress when aggregate unemployment rises [23,24]. Consistent with this, Golberstein et al. [16] find that parental unemployment does not fully explain the relationship between child mental health and economic conditions. The authors conclude that other mechanisms, including increased family stress, are likely to be important pathways through which recessions negatively affect children’s mental health. 

At the same time, the worsening economic conditions that families anticipate during economic downturns are often highly correlated with the real deterioration in economic conditions they experience. Thus, economic recessions may also be linked to poorer child mental health through a range of other mechanisms including: job loss, job instability, low wages, poor work quality, residential moves, diminished parental investments, increased parental stress and lower parenting quality, and marital tension, as well as subjective perceptions of economic hardship [7,25,26]. Studies examining the effects of parental job loss on children’s well-being and achievement find that effects are concentrated in lower socioeconomic-status (SES) households [26]. Recession may also affect children through changes at the community level including reduced expenditure on health and social services, higher unemployment and underemployment rates, aggregate household poverty, and a concentration of disadvantage and negative peer influence [25]. 

As was true across the globe, the onset of the Great Recession in late 2007 resulted in a rapid rise in economic uncertainty and loss of consumer confidence among Australian families [27,28]. However, Australia is unique in avoiding the recession occurring elsewhere with GDP growth remaining strong and unemployment rates rising only two percentage points. The widely anticipated and severe economic downturn in large part did not materialize. The Rudd Government’s AU$42 billion economic stimulus package was passed in the Senate in February 2009 [29]. The stimulus package had three components—a tax bonus for working Australians, a back to school bonus and a single income family bonus for families with children. Cash payments targeted at low income families were effective in stimulating consumption among low-income families with children. 

Little is known about the effects of the Great Recession on child and adolescent mental health [6,9,25,30]. This is important because mental health problems often onset in childhood, are common and burdensome, and left untreated can negatively impact on health, social and economic outcomes into adulthood [31,32]. Although vulnerable adult sub-populations are more negatively affected by actual declines in economic conditions, amongst children the effects may be more universal. In the United States, for example, Golberstein et al. [16] find negative effects of macroeconomic conditions (measured by area unemployment rates and housing prices) on child and adolescent mental health; effects that were pervasive in all population subgroups. Page et al. [17] also found that an increase in local unemployment rates is associated with small but significant increases in severe emotional difficulties among children. There is further evidence that adolescent health may be worse in economic downturns. Job losses stemming from mass layoffs, for example, result in increased adolescent suicidality [33] and more young people seeking emergency psychiatric care [34]. In contrast, in cross-national analyses, Pfoertner et al. [35] found little association between increased country level unemployment rates among adolescents and psychological health before and after the recession. 

Those studies that focus on community perceptions of economic conditions during recessions indicate that sentiment matters as much for child and adolescent outcomes as do actual conditions; particularly for boys. Schneider and colleagues [36] show links between community perceptions of the economic environment during the Great Recession in the United States and child behaviors, high frequency spanking [37], and risks for maternal child abuse and neglect [38]. Schneider et al. [36] find the decline in consumer confidence during the Great Recession, as measured by the Consumer Sentiment Index, to be associated with higher rates of behavioral problems for boys but not girls. These behaviors are aggression, anxiety/depression, alcohol and drug use, and vandalism, with associations largely concentrating in single-parent families. Local unemployment rates, in contrast, display fewer associations with children’s behavior, suggesting that, in the Great Recession, uncertainty about the national economy is the more salient risk for behavior problems rather than local labor markets.

Finally, there are a handful of studies that have examined the effect of the Great Recession on population health in Australia. One study found poorer psychological functioning of older adults who reported to be impacted by the Great Recession [39] with another finding little evidence of impact on mental health problems in adults [40]. In their study of youth pooling four cohorts of the Australian Longitudinal Study of Youth (LSAY), using propensity score matching for control cohorts and difference-in-differences techniques, Parker et al. [41] find significantly lowered well-being across several measures at age 19 with less consistent results at age 22. In part, these measures reflect unhappiness with career and future prospects; however, there were no specific measures of mental health. They further find a decline in well-being from 2011 to 2013 after a recovery in 2010 which mapped to unemployment rates at the time.

Given this context, the objective of this paper is to extend on the work of Golberstein [16], Schneider [36], and further our understanding of the process by which economic conditions, particularly community perceptions of economic uncertainty, affect children’s mental health. We do so by accounting for consumer confidence in addition to actual labor market conditions in our models, using the full SDQ measure (they use an abbreviated five-item measure), and seeking to replicate in an Australian setting. We use longitudinally gathered and richly characterized developmental measures of parents, children, and area-level effects, covering the period before, during, and after the Great Recession in Australia. These data allow us to isolate the mental health effects of the stress generated by anticipation of the Great Recession from any mental health effects associated with deteriorating macroeconomic conditions. Specifically, we ask whether there is an adverse effect on child mental health during the period of the Great Recession associated with local unemployment expectations, whether this differs between boys and girls, and whether there is also an increase in levels of mothers’ distress associated with local unemployment expectations during this time. 

## 2. Materials and Methods 

### 2.1. Data

For this project, we link multiple data sources to construct a data set which includes information on a sample of mothers and their children, local unemployment expectations and local labor market conditions. The data sets, key variables, and linkage process are described below. 

#### 2.1.1. Longitudinal Study of Australian Children

The Longitudinal Study of Australian Children (LSAC) is a national study which was designed to deepen the understanding of child development, in the context of Australia’s social, economic, and cultural environment [42,43,44,45]. The study recruited two cohorts, a birth cohort (comprising 5107 children aged 0–1 years) and a child cohort (4983 children aged 4–5 years), known as cohort B and K, respectively. The LSAC interviews multiple informants; these include the child, the primary caregiver (97 percent of which are the biological mother) and their partner, and the child’s teacher. Interviews for wave one were undertaken in 2004, with follow-up interviews conducted on a bi-annual basis. There are currently six waves of data released, covering the years 2004 to 2014, which allows us to capture the pre- and post-crisis periods. 

Children’s mental health is measured using the Strengths and Difficulties Questionnaire (SDQ). The questionnaire comprises 25 items that collapse into five problem scales: emotional symptoms, conduct problems, hyperactivity-inattention, peer problems, and prosocial behavior [46]. The questions for each sub-scale are the same at each age, other than two questions on the conduct disorders sub-scale which vary slightly for 4-year-olds, with two items on antisocial behavior replaced by items on oppositionality. 

The SDQ total score is a sum of scores on 20 items (omitting prosocial items), with higher scores representing poorer psychosocial functioning. Each item is scored a zero, one, or two based on the scoring key (not true, somewhat true, certainly true), giving a maximum score of 10 for each sub-scale and an SDQ total ranging from zero to 40. A number of items are reverse coded. Where there is missing data, scores are averaged within subscales, so long as there are two or more items answered within the sub-scale. The SDQ total score forms our outcome measure of child mental health because it has been shown to be a psychometrically sound measure of overall child mental health problems [47,48,49,50,51]. 

The SDQ total difficulties score can be disaggregated into two sub-components: internalizing and externalizing difficulties [51]. The internalizing score is the sum of the emotional and peer problems scales, while the externalizing score is the sum of the conduct and hyperactivity scales. Both scores are also increasing in the degree of difficulties the child has. For ease of interpretation, the SDQ total, internalizing, and externalizing scores have been standardized to have a mean of zero and a standard deviation of one.

In addition to the parent-reported SDQ measures, we use the LSAC data to create the SDQ total, internalizing and externalizing scores for children as rated by their teachers. Here, we think that teacher-reported measures might be less subjective than parent-reported measures. While there is a strong correlation between parent- and teacher-rated SDQ scores [52,53], Goodman et al. [48] finds that parents are slightly better at detecting emotional difficulties, while teachers are better at detecting conduct and hyperactivity issues, given they observe the children in different contexts. Thus, as other researchers suggest, there is value in analyzing the responses of multiple informants [53]. However, given the lower survey response rates of teachers we do lose sample size (69% of eligible children have teachers who filled in the survey at wave one). 

Our measure of mothers’ mental well-being is derived from the Kessler Psychological Distress Scale (K6) [54]. The K6 comprises six items on a five-point response scale, which are designed to assess the frequency of distress/depression in the previous four weeks. Items are summed to produce a score that ranges from zero to 24, increasing in the degree of distress. For ease of interpretation, the K6 score has been standardized to have a mean of zero and a standard deviation of one. Although declining labor market conditions are just as likely, if not more likely, to have impacted on fathers, data on K6 were missing for 25 percent of fathers who were present in the household, and thus the data were considered unreliable for analyzing fathers’ distress.

Using the LSAC data we utilize a set of control variables which are motivated by prior research on child and adolescent mental health, with a particular emphasis on socio-demographic determinants of child and adolescent mental health. Our controls comprise the child’s age; the mother’s education level and employment status; family structure (i.e., number of children in the household and presence of two biological parents, one parent, or blended family as an indicator for whether there was a change in household structure since the previous wave); an indicator for whether the family moved house since the previous wave; the log of equivalized real household income; homeownership structure (outright, mortgage, rent, other, missing); a measure of the socio-economic disadvantage of the region (SEIFA) [55]; and area remoteness (major city, inner regional, outer regional, remote). Control variables differ slightly depending on whether the well-being of the child or mother is the outcome of interest. 

Additionally, we utilize a set of local labor market controls to differentiate economic sentiment from actual economic conditions. These include the unemployment rate for males, the unemployment rate for females, the employment to population ratio for males, and the employment to population ratio for females. These variables are derived from the Australian Bureau of Statistics’ (ABS) labor force statistics for local labor market regions (defined as Statistical Area 4—SA4) [56]. 

For a detailed summary and descriptive statistics for all variables see Table S1 in the supplementary material. 

#### 2.1.2. Consumer Attitudes, Sentiments, and Expectations in Australia Survey

The Consumer Attitudes, Sentiments and Expectations in Australia Survey (CASiE), is a monthly telephone survey of 1200 households across Australia, that is conducted by the Melbourne Institute of Applied Economic and Social Research. The CASiE Survey is funded principally by the Westpac Banking Corporation and is supported by contributions from other sources, including the Reserve Bank of Australia. The survey began in 1974 and is modelled after the University of Michigan’s Survey of Consumers. It is aimed at gauging public perceptions of the state of the economy, collecting information on topics such as price and unemployment expectations. These consumer expectations data are particularly valuable for creating indices and forecasting the state of the economy. 

Among other key topics, the CASiE asks each respondent about their unemployment expectations, specifically: 

“Now about people being out of work during the coming 12 months. Do you think there’ll be more unemployment than now, about the same, or less?”

Possible responses include: more unemployment, about the same, less unemployment or don’t know. Based on aggregating and weighting the person-level responses to these questions, we create a monthly unemployment expectations index (UEI) for each labor market region (details on the aggregation of CASiE data provided in Appendix A). This index follows the “balanced approach” which was developed by the University of Michigan and is standard in the expectations literature. It is calculated by taking the proportion of people who say unemployment will increase minus the proportion of people who think it will decrease and adding 100. Thus, the index ranges from zero to 200. An index greater than 100 means a region has pessimistic employment expectations (i.e., expects more unemployment) and an index below 100 means a region is optimistic about employment conditions.

**Figure 1:**
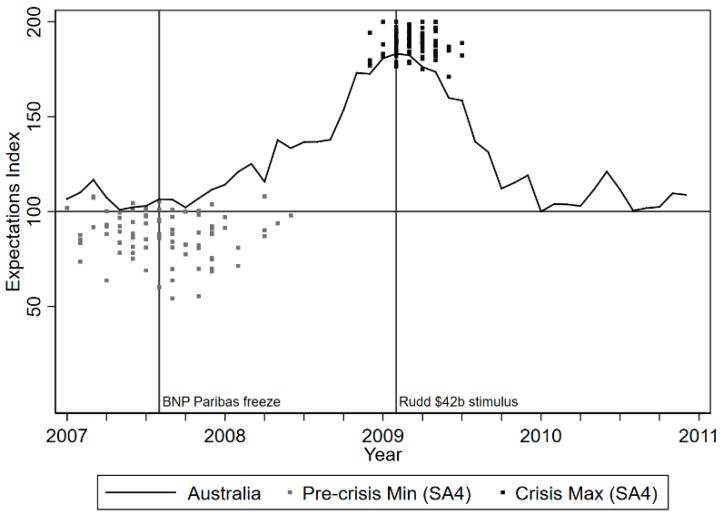
National and community-level unemployment expectations index (UEI) from 2007 to 2011.

The solid line in Figure 1 plots the national unemployment expectations index for Australia over the period 2007 to 2011. It is evident that at the peak of the Great Recession, when Prime Minister Kevin Rudd released a AU$42b stimulus package, that unemployment expectations were also peaking. The index suggests that approximately four in five persons expected unemployment to increase over the next 12 months. Further, Figure 1 also plots the most optimistic expectations (minimum index) prior to the crisis and the most pessimistic expectations during the crisis (maximum index) for each labor market region. The most pessimistic community-level expectations were highly concentrated around the first quarter of 2009, while the most optimistic expectations were more dispersed over 2007–2008. The change in the index between the minimum and maximum represents each community’s “shock to expectations”. The mean change is 100 points, with a standard deviation (SD) of 13. This suggests that there were regions that experienced larger “shocks” to their expectations than others. 

We use this variation in expectations shocks to classify regions into two groups. Specifically, we create a binary variable that equals one if a region experienced a shock in the top 25th percentile of the distribution (i.e., a very intense shock to expectations over the crisis period), and zero otherwise. The mean change for the treated is 118.43 points, the mean change for the untreated is 94.96 points. This unemployment “expectations shocks” is a key variable in our difference-in-differences estimation strategy to be described below. In addition, we create a less intense measure of “expectations shocks”, which equals one if the region experienced an unemployment expectations shock greater than the average and zero otherwise, to be used in sensitivity analyses.

### 2.2. Data Linkage

We link our data sets together using a geographic measure defined by the ABS; Statistical Area Level 4 (SA4s) [57]. Given our focus on unemployment expectations, SA4s are particularly relevant because they are reflective of labor markets within each state and territory. Regional SA4s typically have 100,000 to 300,000 persons, while metropolitan SA4s have up to 500,000 persons. There is a total of 107 SA4s in Australia. Our sample covers 83 SA4s; we exclude 19 SA4s with the classification of migratory—offshore—shipping or no usual address and five SA4s with unavailable CASiE data. 

We merge the CASiE data to LSAC observations using the respondents’ SA4 of residency. Since our expectations shock variable does not change over time we do not need to merge on a time dimension. However, we merge ABS data to each LSAC observation using each respondents’ SA4 of residency and the month/year of interview, to capture local labor market conditions at the time of reporting. 

### 2.3. Analysis Sample

For our analysis, we draw on data from the 4983 children in cohort K and focus on the unbalanced panel using waves one, two and four (13,616 observations) (see Table S2 in the supplementary material). Wave three (2008) is excluded because child outcomes might have already been influenced by the Great Recession throughout 2008 and waves five and six are excluded because outcomes are likely influenced by the Euro Crisis. We drop observations which are missing data for the key variables in our analysis: the expectations shock (424 observations, 3.11 percent of observations), parent-reported SDQ measures (279 observations, 2.04 percent) and control variables (125 observations, 0.92 percent). The resulting sample includes 4862 children (12,788 observations). Finally, part of our estimation strategy (explained below) requires that children be present in both waves one and two. This reduces our analysis sample to 4089 children (11,694 observations), which is mostly driven by sample attrition. At waves two and four, children are aged 6–7 years and 10–11 years old respectively.

A similar method is used to select the sample of mothers for the analysis of mother’s psychological distress (see Table S3 in the supplementary material). Our base sample consists of the 4853 primary female guardians of the children in cohort K, over waves one, two, and four (13,178 observations). We exclude observations which have missing data for the expectations shock (400 observations, 3.03 percent of observations), psychological distress (1069 observations, 8.11 percent) and control variables (443 observations, 3.36 percent). Again, we restrict the sample to mothers who were present in both waves one and two. The final analysis sample consists of 3321 mothers (9562 observations), 99.7 percent of which are biological. A total of 68.4% of mothers are retained for the analysis sample. 

### 2.4. Statistical Analysis

#### 2.4.1. The Difference-In-Differences (DID) Estimator 

Our statistical analysis involves estimating difference-in-differences (DID) models to assess the impact of community-level unemployment expectations shocks over the Great Recession period on children’s mental health (SDQ) and mothers’ psychological distress (K6). 

The timing of the Great Recession and LSAC interviews inform our choice of pre- and post-crisis periods (described in detail in Appendix B). We focus on waves one and two (2004 and 2006) as the pre-crisis periods and wave four (2010) as the post-crisis period. The expectations shocks variable allows us to separate regions into those who experienced an intense change in unemployment expectations due to the Great Recession (top 25th percentile) and those who did not. We will refer to these communities as experiencing “expectations shocks”. In essence, the DID model compares the pre- and post-crisis outcomes of children who live in communities that experienced expectations shocks (treatment group) to those that did not experience them (control group). 

In formalizing the DID model, we estimate the following equation:

(1) $$SDQ_{irt}=\beta_{0}+\beta_{1}post_{t}+\beta_{2}shock_{r}+\beta_{3}\left(post_{t}\times shock_{r}\right)+X_{irt}^{\prime }\gamma +e_{irt}$$

where $SDQ_{irt}$ is the SDQ outcome for child *i*, in region *r*, at time *t*, $post_{t}$ is a binary variable which equals one if the LSAC wave is post crisis, $shock_{r}$ is the binary unemployment expectations shock variable, $post_{t}\times shock_{r}$ is an interaction of the two. Meanwhile, $X_{irt}^{\prime }$ is a vector of demographic and geographic control variables and $e_{irt}$ is the error term. 

In our model, $\beta_{0}$ estimates an overall “intercept”, $\beta_{1}$ estimates the average change in the SDQ scores from the pre- to post-crisis periods for the control group, and $\beta_{2}$ estimates the average difference in SDQ scores between treatment and control groups prior to the GFC. The coefficient for the interaction term, $\beta_{3}$ (also known as the DID coefficient), captures the differential effect of the Great Recession on children’s mental health in communities that experienced a large shock in unemployment expectations versus in those communities that did not. That is, $\beta_{3}$ addresses the study question of whether community perceptions of macroeconomic conditions during the Great Recession had a causal impact on children’s mental health.

In the handful of studies that examined differences in macroeconomic conditions and child mental health, findings by child gender are mixed [16,38]. Therefore, we estimate Equation (1) separately by child sex to assess whether boys and girls respond differently to changes in unemployment expectations.

Further, there may be concerns that parent-rated measures might be reflective of the mental well-being of the parent themselves [16]. One strength of the LSAC data is that the child’s teachers also complete the SDQ. This gives us the opportunity to use a potentially less subjective measure of child mental health as an alternative outcome. 

We also want to assess if the changes in unemployment expectations affect the psychological distress of mothers. To do so, we re-estimate Equation (1) with the mother’s standardized K6 score as the outcome variable. Also, note that the explanatory variables for this model are slightly changed to reflect that the person of interest is now the mother, rather than the child. 

Given our focus on expectations shocks, we wish to examine the extent to which the expectations of a crisis influence children’s and mother’s mental well-being independent of actual labor market conditions. Even though Australia mostly avoided the effects of the Great Recession, it is evident that some local labor markets were hit harder than others. For example, in 2009 across local labor markets unemployment rates ranged from one to 17 percentage points [56]. Thus, we estimate Equation (1), for children and mothers, with and without local labor market controls. 

#### 2.4.2. Identification

Equation (1) captures the causal effect of a worsening in economic expectations on children’s mental health so long as the time trend in children’s mental health is the same in communities that do and do not experience expectations shocks. That is, the trends in mental health should be the same in the absence of any treatment. This is the so-called parallel trends assumption [58].

To verify this assumption, we test for statistical differences in pre-crisis SDQ scores between treatment and control groups. Using unadjusted linear regression models where the outcome is the change in the SDQ variable (between waves one and two) and our binary expectations shocks variable is the only explanatory variable, we verify the parallel trends assumption is reasonable. That is, prior to the Great Recession the children in communities that did and did not experience expectations shocks have similar trends in their outcomes (see Table S4 in the supplementary material). Further, the parallel trends assumption holds for boys and girls, separately. For mothers, the assumption does not hold, but does hold when using our alternative definition of expectations shocks (i.e., unemployment expectations shocks greater than the average) (see Table S5 in the supplementary material). 

## 3. Results

### 3.1. The Effects of Unemployment Expectation Shocks on Parent-Rated SDQ Outcomes

Table 1 presents the results for the effects of unemployment expectations shocks (in the top 25th percentile) on parent-rated standardized SDQ outcomes for boys and girls (Panels A and B respectively). We estimate two sets of models, columns (1)–(3) exclude local labor market controls and columns (4)–(6) include them. The DID coefficient shows that, on average, there is no significant difference in boys’ parent-rated SDQ (total, internalizing, and externalizing) scores as a result of the Great Recession, regardless of whether local labor market conditions are controlled for. This suggests that boys’ mental well-being is not affected by unemployment expectations shocks. It is worth noting that, on average, the boys in communities that experience larger expectations shocks have more difficulties and externalizing behaviors prior to the crisis. For girls, there is a modest increase in the SDQ total (0.133 of a standard deviation) and SDQ externalizing scales (0.128 SD). That is, the difference in levels of mental health problems and externalizing problems pre- and post-crisis is greater among girls living in regions that experience an intense unemployment expectations shock during the crisis. The result is stable and slightly larger after controlling for local labor market conditions, suggesting that unemployment expectations affect the mental well-being of girls independent of objective unemployment conditions. 

### 3.2. The Effects of Unemployment Expectation Shocks on Teacher-Rated SDQ Outcomes

Given concerns that parent-rated measures might be reflective of the mental well-being of the parent themselves, we replicate Table 1 replacing parent-rated SDQ measures with teacher-rated measures. Table 2 presents the results for boys and girls. Consistent with the parent-rated measures, we find no significant effects of changes unemployment expectations on boys’ SDQ scales (Panel A). For girls, when using the teacher-rated SDQ outcomes we also find no significant difference in SDQ scores caused by unemployment expectations shocks (Panel B). 

### 3.3. The Effects of A Greater than Average Change in Unemployment Expectation Shocks on Parent and Teacher-Rated SDQ Outcomes

To test the sensitivity of our child results to our definition of expectations shocks, we replicate the results using a less intense measure. Specifically, expectations shocks are now defined to be ‘a change in unemployment expectations over crisis period greater than the average change’, rather than a change in unemployment expectations in the top 25th percentile. Table 3 presents the results for boys and girls, for both parent- and teacher-reported outcomes and includes the labor market controls. Using a less intense measure of shocks does not change our conclusions for boys (Panel A). That is, they still appear to be emotionally unresponsive to changes in unemployment expectations. For girls, we continue to find no effects, on average, when using teacher-rated measures (Panel B). Perhaps unsurprisingly, the modest effect sizes previously found for girls, when using parent-reported outcomes, are smaller when using the less intense measure of shocks. For example, the increase in SDQ total problems is reduced by almost half, from 0.152 to 0.089 of a standard deviation. A similar result is found for externalizing problems. Additionally, these estimates are less precise. 

### 3.4. The Effects of Unemployment Expectations Shocks on Mother’s Psychological Distress

Next, we turn our attention to the mothers of the children to determine if their psychological distress is affected by the changes to unemployment expectations over the Great Recession period. In Table 4 we present estimates for the difference-in-differences models of mother’s standardized Kessler 6 score. The results in Panel A suggest that, on average, the levels of psychological distress are no different as a result of the Great Recession between mothers living in areas that experienced an unemployment “expectations shock” in the top 25th percentile to those living in areas with more mild shocks. This result is independent of the actual local labor market conditions. We should, however, interpret this result with caution given that the parallel trends assumption does not hold for this treatment variable. That is, there were already differences in the trends of psychological distress prior to the crisis between mothers in the treatment and control groups. Interestingly, post-crisis all mothers experience elevated levels of distress by approximately a 10th of a standard deviation. Panel B repeats the analysis for mothers using the alternative definition of “expectations shocks” (i.e., greater than the average shock), for which the parallel trends assumption holds, and the results are largely unchanged. 

## 4. Discussion

In this paper, our goal is to shed light on the impact of the Great Recession on child mental health. We exploit the unique Australian circumstance, where a crisis did not eventuate, to differentiate economic conditions from shocks in community-level expectations about local economic conditions. Overall, we find a story of very little association between shock to unemployment expectations and child mental health outcomes or mothers’ psychological distress. For boys, there is no detectable effect of expectations shocks on mental health. For girls, there are modest effects on total SDQ and the externalizing sub-scale when using parent-rated but not teacher-rated outcomes. These results are stable after controlling for actual labor market conditions. 

It is reassuring that despite significant changes in community-level economic sentiment in Australia, children’s mental health is mostly unaffected. The treatment group communities have shocks to unemployment expectations that are 25 percent higher than those in the control groups. During peak crisis times, on average, nine in ten persons in these communities expected unemployment would increase in the next 12 months. Despite this, expectations shocks do not appear to affect child mental health from pre- to post-crisis periods (between 2006 and 2010). Where there is a treatment effect, it is notably modest in effect size. 

Within the Australian context, there is only one study that we are aware of amongst youth with which to compare these results. During the Great Recession period, Parker et al. [41] found lower well-being across several measures at age 19 including career and future prospects, which perhaps maps more closely to the unemployment expectations index (UEI) than the mental health measures in our study. In Australia, the shock to unemployment expectations dissipated dramatically after the introduction of the stimulus package in February 2009, which may have meant that fear and stress within families associated with expected worsening job conditions and potential layoffs quickly subsided, easing any effects of perceived economic hardship on children. Perhaps unsurprisingly, countries with strong social safety nets and those that introduced budgetary stimulus seemingly buffered some of the negative effects on adult mental health [9,15,59].

Previous findings on the mental health consequences of poor macroeconomic conditions are based on only a handful of studies from the United States, where the economic crisis hit harder. Studies based on actual area level economic conditions (e.g., unemployment rates, housing prices, layoffs) report an increase in children’s mental health problems [16,17], suicidal behaviors in adolescents [33] and in youth seeking emergency psychiatric care [34]. Further evidence from the United States demonstrated that community fear and uncertainty about national economic conditions during the Great Recession independently predicted behavioral and emotional problems amongst 9-year-old boys, not girls, more consistently than local labor market conditions [36]. These significant effects were concentrated in single-parent families and partially explained by parenting behaviors. Part of the reason we find no substantial effects on child mental health, in addition to the country setting, may be the timing of outcome measurement. Although our study captures a similar age group, Schneider et al. covered the period before and during the crisis (2007–2010), whereas we measure changes in child mental health before and after the peak. The effects on the family environment, such as via parental distress or more undesirable parenting practices, may be more temporal, especially given the short reference period for the SDQ (last six months).

In our analyses, living in regions experiencing an expectations shock is associated with increased externalizing behaviors based on parent-rated SDQ for girls but not boys. As previous studies have produced mixed findings about the effects of macro-economic conditions (real or perceived) on the mental health of boys and girls, we could not hypothesize stronger effects either way. While past work suggests girls’ mental health may be more susceptible to actual economic conditions [16,60], worsening consumer confidence in the United States during the Great Recession was associated with an increased in emotional and behavioral problems among boys not girls [36]. More generally, in their review of moderating effects between stressors and psychopathology, Grant and colleagues [61] reported that boys tend to respond to stressors with externalizing symptoms while girls respond more with internalizing symptoms; this is particularly the case for studies of poverty, divorce, and abuse.

In contrast to models using parent-rated SDQ, we find no significant effect among girls (or boys) based on teacher-rated SDQ. The parent- and teacher-rated SDQ is known to yield different results in terms of identifying mental health problems in children [62]. This is partly explained by the different behaviors observed by parents in the context of their own home and the relationship with their children, compared to what might be a different set of behaviors observed by teachers in the classroom setting. Furthermore, examining changes in mental health over time using teacher-rated SDQ is also subject to lower reliability due to the potential change of teacher over time. Additionally, we have a reduced sample size for teacher versus parent assessments.

We do not find any association between unemployment expectations shocks and maternal psychological distress, which we considered as a potential mechanism by which shocks could be transmitted from parents to children. One explanation may be the limited reference period for experiencing symptoms (four weeks) as measured by the Kessler 6 which may not have captured elevated stress levels caused by the shock to expectations during the peak of the crisis. Other work on mechanisms has considered the impact on parenting. Studies have found negative effects of the Great Recession on parenting in the form of more spanking [37] and increased levels of neglect and abuse [18]. We had insufficient data to consider father distress. The global economic downturn is generally associated with a larger decline in mental health for men than for women, particularly among men of working ages [8] although increased rates of anxiety have been observed amongst low income and unemployed women in the United States [63]. 

### Strengths and Limitations

This study has several strengths. First, the Australian economic setting through the Great Recession was unique in so far as the shock was largely contained to a perceived global threat rather than an actual economic crisis. Second, the LSAC data capture a large cohort of Australian children that is nationally representative for Australia and who lived contemporaneously through this event. Third, the data afford a unique data linkage of the LSAC cohort with the CASiE data covering pre-, mid-, and post-recession periods in Australia. In doing so, we combine the rich contextual information of LSAC with a community-level set of economic sentiments. Fourth, the further linkage of LSAC data with ABS labor force statistics allows us to examine the specific effect of community-level sentiment without economic conditions confounding the results, which is a particular feature of the Australian Great Recession experience. We also use an internationally validated measure of emotional and behavioral problems in the SDQ, and in comparison to Golberstein et al. [16] who were limited to an abbreviated version, the LSAC administers the full instrument at each wave. Finally, by using a difference-in-differences methodology with longitudinal data we find estimates that are more plausibly causal.

There are also limitations. First, we have mostly a story of null findings. Care needs to be taken in interpreting these null findings; we do not wish to conflate a lack of statistical significance with proof of non-existence [64]. In contrasting boys vs. girls, parent vs. teacher we take care to not over-interpret our findings. We have not performed pairwise comparisons, and we are not interested in contrasting significant and non-significant effects [65]. 

The LSAC largely uses brief measures. While the SDQ [47,48,49,50,51] and Kessler 6 [54] are well regarded screening scales, these measures do not detect psychiatric caseness as well as more extensive tests such as the World Health Organization’s Composite International Diagnostic Interview (CIDI) [66]. Thus, despite the large sample size, we need to be aware that measurement error biases our findings towards the null [67].

Further, in the model of mothers’ psychological distress, the parallel trends assumption is violated in some cases indicating that those mothers living in areas that experienced intense expectations shocks were on a different trajectory of distress compared to those who did not experience a shock. This means that the results for these models should be interpreted with caution.

## 5. Conclusions

Our study provides a unique examination of the mental health effects of the Great Recession as experienced in Australia with a focus on the sharp rise in pessimistic employment expectations during the peak of the crisis. We find little effect which could have been due to the timely and seemingly effective response of the Australian Government in protecting the economy or due to other reasons. 

Our key research finding was an impact for girls but not boys. This is a population-level effect and we do not fully understand the mechanisms via which girls’ mental health is affected, or whether the effects are stronger in vulnerable sub-groups of the population (e.g., low-income families who were targeted by the Government’s stimulus package). Further research applying individual and family-level analyses may be able to give us a better understanding of the potential pathways and the role of coping mechanisms that do or do not allow economic stress to impact on children and their mothers. 

## Figures and Tables

**Table 1:** The effects of unemployment expectations shocks (top 25th percentile) on parent-rated SDQ outcomes, separately for boys and girls.

	Models Excluding Local Labor Market Controls			Models Including Local Labor Market Controls		
	SDQ Total	Internalizing	Externalizing	SDQ Total	Internalizing	Externalizing
	(1)	(2)	(3)	(4)	(5)	(6)
**A: Boys**						
Expectations shock	0.082 **	0.021	0.108 ***	0.069 *	0.016	0.092 **
	(0.040)	(0.040)	(0.040)	(0.041)	(0.042)	(0.041)
Post-crisis	−0.056	−0.010	−0.076	−0.044	0.002	−0.068
	(0.127)	(0.128)	(0.127)	(0.128)	(0.128)	(0.127)
DID	0.002	−0.030	0.027	−0.002	−0.034	0.024
	(0.071)	(0.072)	(0.071)	(0.072)	(0.072)	(0.072)
$H_{1}$: DID > 0 (*p*-value)	0.491	0.663	0.352	0.513	0.682	0.368
R-Square	0.083	0.057	0.076	0.084	0.058	0.077
*N*	5960	5960	5960	5960	5960	5960
**B: Girls**						
Expectations shock	−0.026	−0.014	−0.029	−0.042	−0.034	−0.035
	(0.037)	(0.039)	(0.036)	(0.038)	(0.040)	(0.037)
Post-crisis	0.034	0.194	−0.106	0.044	0.209 *	−0.103
	(0.113)	(0.119)	(0.112)	(0.113)	(0.119)	(0.112)
DID	0.133 **	0.091	0.128 **	0.152 **	0.108	0.143 **
	(0.066)	(0.069)	(0.065)	(0.066)	(0.070)	(0.065)
$H_{1}$: DID > 0 (*p*-value)	0.021 **	0.094 *	0.024 **	0.011 **	0.060 *	0.015 **
R-Square	0.108	0.062	0.113	0.110	0.063	0.113
*N*	5734	5734	5734	5734	5734	5734

Notes: Estimated Ordinary Least Squares (OLS) coefficients are presented with standard errors in parentheses. All models control for demographic, financial, and regional variables, unless otherwise indicated. Complete regression results can be found in Table S6 for boys and Table S7 for girls. *, **, *** indicate significance at the 10%, 5%, and 1% levels, respectively. DID: difference-in-differences.

**Table 2:** The effects of unemployment expectations shocks (top 25th percentile) on teacher-rated SDQ outcomes, separately for boys and girls.

	SDQ Total	Internalizing	Externalizing
	(1)	(2)	(3)
**A: Boys**			
Expectations shock	0.014	−0.007	0.024
	(0.051)	(0.049)	(0.052)
Post-crisis	−0.410 ***	−0.361 **	−0.322 **
	(0.154)	(0.150)	(0.156)
DID	0.062	0.076	0.029
	(0.085)	(0.083)	(0.086)
$H_{1}$: DID > 0 (*p*-value)	0.235	0.177	0.368
R-Square	0.047	0.030	0.038
*N*	4500	4502	4502
**B: Girls**			
Expectations shock	0.036	−0.010	0.059
	(0.041)	(0.047)	(0.039)
Post-crisis	−0.053	0.131	−0.173
	(0.120)	(0.136)	(0.112)
DID	0.019	0.001	0.032
	(0.069)	(0.079)	(0.065)
$H_{1}$: DID > 0 (*p*-value)	0.393	0.496	0.310
R-Square	0.065	0.036	0.055
*N*	4394	4394	4397

Notes: Estimated OLS coefficients are presented with standard errors in parentheses. All models control for demographic, financial, regional, and local labor market controls, which is our preferred specification. *, **, *** indicate significance at the 10%, 5%, and 1% levels, respectively.

**Table 3:** The effects of less intense unemployment expectations shocks (greater than average) on parent- and teacher-rated SDQ outcomes, separately for boys and girls.

	Parent-Rated			Teacher-Rated		
	SDQ Total	Internalizing	Externalizing	SDQ Total	Internalizing	Externalizing
	(1)	(2)	(3)	(4)	(5)	(6)
**A: Boys**						
Expectations shock	0.027	−0.034	0.068 **	0.033	−0.001	0.046
	(0.033)	(0.034)	(0.033)	(0.041)	(0.040)	(0.041)
Post-crisis	−0.033	0.001	−0.052	−0.385 **	−0.347 **	−0.300 *
	(0.129)	(0.129)	(0.128)	(0.156)	(0.151)	(0.158)
DID	−0.023	−0.032	−0.010	−0.027	−0.003	−0.035
	(0.058)	(0.058)	(0.058)	(0.069)	(0.067)	(0.070)
$H_{1}$: DID > 0 (*p*-value)	0.655	0.706	0.566	0.654	0.518	0.689
R-Square	0.083	0.058	0.076	0.047	0.030	0.044
*N*	5960	5960	5960	4500	4502	4502
**B: Girls**						
Expectations shock	−0.066 **	−0.047	−0.062 **	0.002	0.001	0.003
	(0.030)	(0.032)	(0.030)	(0.033)	(0.037)	(0.030)
Post-crisis	0.034	0.203 *	−0.112	−0.078	0.108	−0.202 *
	(0.114)	(0.121)	(0.113)	(0.121)	(0.137)	(0.113)
DID	0.089 *	0.062	0.085	0.061	0.056	0.045
	(0.052)	(0.055)	(0.052)	(0.055)	(0.062)	(0.051)
$H_{1}$: DID > 0 (*p*-value)	0.043 **	0.129	0.050 **	0.135	0.183	0.190
R-Square	0.110	0.063	0.113	0.065	0.036	0.060
N	5734	5734	5734	4394	4394	4397

Notes: Estimated OLS coefficients are presented with standard errors in parentheses. All models control for demographic, financial, regional, and local labor market controls, which is our preferred specification. *, **, *** indicate significance at the 10%, 5%, and 1% levels, respectively.

**Table 4:** The effects of unemployment expectations shocks on mother’s psychological distress score (Kessler 6).

	Model Excluding Local Labor Market Controls	Model Including Local Labor Market Controls
**A: “Expectations shock”: top 25th percentile**		
Expectations shock	0.061 **	0.077 **
	(0.031)	(0.032)
Post-crisis	0.104 ***	0.098 ***
	(0.028)	(0.029)
DID	−0.069	−0.074
	(0.055)	(0.056)
$H_{1}$: DID > 0 (*p*-value)	0.893	0.909
R-Square	0.061	0.062
*N*	9562	9562
**B: “Expectations shock”: >average**		
Expectations shock	−0.022	−0.018
	(0.025)	(0.026)
Post-crisis	0.092 ***	0.087 ***
	(0.032)	(0.032)
DID	−0.004	−0.006
	(0.044)	(0.044)
$H_{1}$: DID > 0 (*p*-value)	0.534	0.555
R-Square	0.061	0.061
*N*	9562	9562

Notes: Estimated OLS coefficients are presented with standard errors in parentheses. All models control for demographic, financial, and regional variables, unless otherwise indicated. Complete regression results can be found in Table S8. *, **, *** indicate significance at the 10%, 5%, and 1% levels respectively.

**Figure A1:**
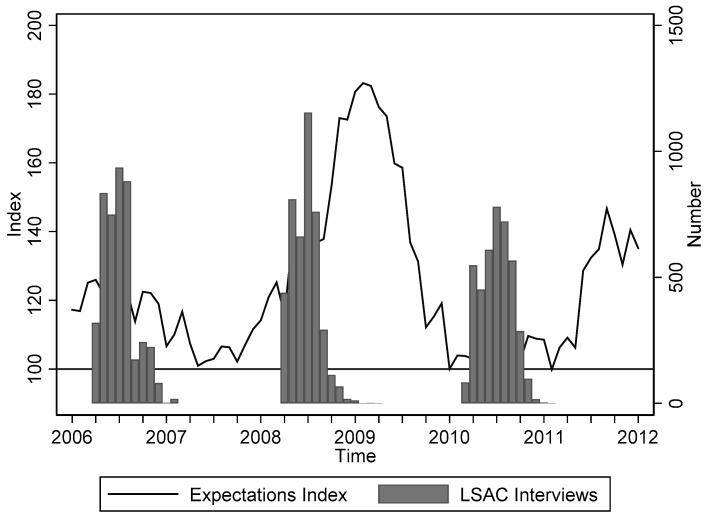
National unemployment expectations index (UEI) and the LSAC interviews, from 2006 to 2012.

## Supplementary Materials

The following are available online at http://www.mdpi.com/1660-4601/16/4/537/s1. Table S1: Variable definitions and summary statistics; Table S2: Creating the analysis sample for children; Table S3: Creating the analysis sample for mothers; Table S4: Tests of parallel trends between treatment and control, children; Table S5: Tests of parallel trends between treatment and control, mothers; Table S6: Complete results for the unemployment expectations shocks (top 25th percentile) on parent-rated SDQ outcomes, boys; Table S7: Complete results for the effects of unemployment expectations shocks (top 25th percentile) on parent-rated SDQ outcomes, girls; Table S8: Complete results for the effects of unemployment expectations shocks on mother’s psychological distress (Kessler 6).

## Data Sharing Statement

This paper uses unit record data from Growing Up in Australia, the Longitudinal Study of Australian Children. The study is conducted in partnership between the Department of Social Services (DSS), the Australian Institute of Family Studies (AIFS). and the Australian Bureau of Statistics (ABS). The findings and views reported in this paper are those of the author and should not be attributed to DSS, AIFS or the ABS. Data are available from the Longitudinal Study of Australian Children authors. They are contactable via: https://growingupinaustralia.gov.au/data-and-documentation/accessing-lsac-data. Users may apply for individual or organizational licenses. There is a fee for the licenses and release of data. The data analyzed in this project were accessed via an organizational license. This paper uses unit record data from a Survey of Consumer Attitudes, Sentiments and Expectations (CASiE) in Australia. Data are available from the Melbourne Institute: Applied Economic & Social Research. The data analyzed in this project can be acquired via an organizational license, which involves a fee. For more information please see: https://melbourneinstitute.unimelb.edu.au/casie.

## Appendix A. Aggregation of CASiE Data

The CASiE data interviews 1200 random people each month in each year for the period 2003–current. The data contains each person’s unemployment expectations about the 12 months ahead, residential postcode and population weight (based on age, gender and location). Using correspondence files provided by the Australian Bureau of Statistics, we match each person’s postcode to their Statistical Area 4 (SA4), which are larger than postcodes and represent local labor markets. Using the population weights and the SA4 data allows us to aggregate person-level responses to an expectations index that is a more accurate representation of unemployment expectations in each local labor market. The process is described in detail below.

The process for creating the monthly unemployment expectations index for each SA4 is as follows:

1. Each CASiE respondent answers the unemployment expectations question by selecting one out of four mutually exclusive categories: More unemployment; About the same; Less unemployment; Don’t know. For each category we generate a binary variable that equals one if the respondent chose those expectations and zero otherwise. Each person’s response is then weighted by their population weight.
2. We need to aggregate the individual-level data to an area-level SA4, so for each SA4 we sum the number of (weighted) responses in each category. We then divide the sum of the (weighted) responses by the sum of the total number of (weighted) respondents in a given SA4. This produces the (weighted) proportion of the SA4′s population who has expectations in each of the four categories (e.g., the (weighted) proportion of the SA4 who believe there will be ‘more unemployment’ over the next 12 months) for each SA4-month combination.
3. There are 85 SA4s covered in the CASiE data, so aggregating over one month of data to produce a monthly unemployment expectation means that we are aggregating over an average of 13.6 CASiE respondents per SA4. The concern here is that the sample for each SA4 is too small to be representative (even with the weights). Thus, for each SA4-month observation, we aggregate expectations over the current month and previous two months.
4. The next step is to create the Unemployment Expectations Index for each SA4. To calculate the index for each SA4-month, we take the (weighted) proportion of people who think there will be more unemployment minus the (weighted) proportion of people who think there will less and plus 100. Thus, the index ranges from zero to 200. An index greater than 100 means a region has pessimistic unemployment expectations and an index below 100 means a region is optimistic about employment conditions.

## Appendix B. Pre- and Post-Crisis Periods

Here we describe the timing of the unemployment expectations and LSAC interviews to determine the pre- and post-Great Recession periods. Figure A1 graphs the Australian unemployment expectations index (left axis) and the number of LSAC interviews across time (right axis), which represents each wave’s interview distribution. Here we focus on wave two (2006) to wave four (2010) interviews. Wave two interviews take place pre-crisis, wave three coincides with the beginning of the crisis, while wave four interviews take place when expectations return to a neutral point, before the Euro Crisis leads to another increase in the unemployment expectations during 2011. From the graph, we can identify waves one and two as pre-crisis periods and wave four as the post-crisis period. We choose to drop wave three because outcomes might already be influenced by the beginning crisis (rising expectations, but not the full impact of the Great Recession), and wave five and six because it might capture the Euro Crisis, cofounded with any remaining Great Recession effects. Thus, we only use waves one, two, and four for the analysis.
