# Supplementary material for: The Great Recession and Children’s Mental Health in Australia
Source: Int J Environ Res Public Health. 2019 Feb 13;16(4):537. doi: 10.3390/ijerph16040537 (PMC6406973; doi:10.3390/ijerph16040537)
Supplement: Supplementary file 1 [file ijerph-16-00537-s001.pdf]

Supporting Information

Table S1. Variable definitions and summary statistics

Variable name	Definition	Mean (SD) Child sample	Mean (SD) Mother sample
**<u>Outcomes</u>**			
SDQ total	The strengths and difficulties questionnaire (SDQ) comprises 25 items that collapse into five problem scales (emotional; conduct; hyperactivity; peer; prosocial). The total difficulties score is generated by summing scores from all the scales except the prosocial scale. The resultant score ranges from zero to 40. For ease of interpretation, the total score has been standardized to have a mean of zero and a standard deviation of one.	0 (1)	-
SDQ Internalizing	The internalizing score ranges from zero to 20 and is the sum of the emotional and peer problems scales. For ease of interpretation, the internalizing score has been standardized to have a mean of zero and a standard deviation of one.	0 (1)	-
SDQ Externalizing	The externalizing score ranges from zero to 20 and is the sum of the conduct and hyperactivity scales. For ease of interpretation, the externalizing score has been standardized to have a mean of zero and a standard deviation of one.	0 (1)	-
Kessler 6	The Kessler 6 (K6) comprises six items on a five-point response scale, which assess the frequency of distress / depression in the previous four weeks. Items are summed to produce a score that ranges from zero to 24. For ease of interpretation, the K6 score has been standardized to have a mean of zero and a standard deviation of one.	-	0 (1)
**<u>Expectations Shocks (CASiE)</u>**			
We use the CASiE data to develop a monthly Unemployment Expectations Index for each Statistical Area 4 (SA4), based on weighting and aggregating person-level CASiE respondents. This index is based on the "balanced approach" which is standard in the expectations literature and was developed by the University of Michigan (also the creators of the consumer sentiment survey). The index ranges from zero to 200. An index >100 means a region has pessimistic unemployment expectations and an index <100 means a region has optimistic unemployment expectations. From this monthly index, for each SA4, we find the difference between the minimum score (more optimistic) prior to the Great Recession and the maximum score during the Great Recession (pessimistic).			
Expectations Shock	Equals 1 if an SA4's min-to-max change is in the top 25th percentile across all SA4s (i.e., experiences a more intense expectation shock).	0.197 (0.397)	0.193 (0.394)
Alternative Expectations Shock	Equals 1 if an SA4's min-to-max change is greater than the average min-to-max change across all SA4s (i.e., a larger than average expectations shock).	0.405 (0.491)	0.406 (0.491)
<u>LSAC Controls</u>			
Child's age	Child's age in years.	6.763 (2.538)	-

Mother’s age	Mother’s age in years.	-	37.632<br>(5.613)
Number of children	Number of children aged less than 18 in the household.	2.515<br>(1.004)	2.495<br>(0.962)
Lone parent	Equals 1 if child resides in a lone-parent household (reference category: two biological parents present).	0.151<br>(0.358)	0.134<br>(0.340)
Blended family	Equals 1 if child resides in a blended family (e.g., step parent, adopted parents, aunts/uncles, grandparents) (ref cat: two biological parents present).	0.035<br>(0.183)	0.028<br>(0.164)
Change in household	Equals 1 if there was any change at all in household composition between waves. For wave one, this information is missing so set the variable equal to zero and use the wave one missing dummy to account for this.	0.155<br>(0.362)	0.143<br>(0.350)
Moved home	Equals 1 if moved in past two years. For wave one, this information is missing so set the variable equal to zero and use the wave one missing dummy to account for this.	0.152<br>(0.359)	0.144<br>(0.351)
Ln equiv.household weekly income	Log of real equivalized household weekly income (at 2010 prices) with missing values imputed where possible and missing/negative incomes set to $1. The equivalence scale used is the OECD modified scale (which assigns a weight of 1 to the first adult in the household, 0.5 for each other adult, and 0.3 for each child). For wave one, this information is missing so set the variable equal to zero and use the wave one missing dummy to account for this.	4.092<br>(3.236)	4.136<br>(3.237)
Imputation flag	Equals 1 if household income is imputed.	0.038<br>(0.192)	0.037<br>(0.188)
Income missing	Equals 1 if household income is missing and was not imputed.	0.030<br>(0.170)	0.028<br>(0.163)
Home: Mortgage	Equals 1 if home is owned with a mortgage (reference category: home is owned outright).	0.620<br>(0.486)	0.640<br>(0.480)
Home: Renting	Equals 1 if home is rented (ref cat: home is owned outright).	0.221<br>(0.415)	0.199<br>(0.399)
Home: Other	Equals 1 if home is in a rent-buy scheme or life tenure scheme etc. (ref cat: home is owned outright).	0.030<br>(0.170)	0.028<br>(0.165)
SEIFA	Decile of index of relative socio-economic disadvantage for regions, where one represents highest relative disadvantage and 10 highest relative advantage (ABS 2001). It takes into account variables such as the proportion of families with high incomes, people with a tertiary education, and people employed in a skilled occupation.	4.936<br>(2.568)	4.999<br>(2.546)
Inner regional	Equals 1 if respondent lives in inner regional Australia (as defined in the Australian Standard Geographical Classification [ASGC]) (reference category: Major urban).	0.206<br>(0.405)	0.208<br>(0.406)
Outer regional	Equals 1 if respondent lives in outer regional Australia (ref cat: Major urban).	0.105<br>(0.307)	0.104<br>(0.305)
Remote	Equals 1 if respondent lives in remote or very remote location in Australia. (ref cat: Major urban)	0.014<br>(0.118)	0.015<br>(0.121)
Wave 1 missing	Equals 1 if wave one information is missing on the following variables: Change in household; Moved home; Ln equiv.household weekly income.	0.350<br>(0.477)	0.347<br>(0.476)
Year 12	Equals 1 if the mother’s highest level of education is the completion of year 12 (reference category: year 11 or below).	0.132<br>(0.338)	0.132<br>(0.339)

Certificate	Equals 1 if the mother's highest level of education is the completion of a certificate (ref cat: year 11 or below).	0.289<br>(0.453)	0.278<br>(0.448)
Diploma	Equals 1 if the mother's highest level of education is the completion of a diploma or advanced diploma (ref cat: year 11 or below).	0.094<br>(0.293)	0.099<br>(0.299)
Bachelor or above	Equals 1 if the mother's highest level of education is the completion of a bachelors or higher (reference category: year 11 or below).	0.311<br>(0.463)	0.325<br>(0.468)
Employed	Equals 1 if the mother is currently employed (reference category: not in the labor force).	0.676<br>(0.438)	0.690<br>(0.462)
Unemployed	Equals 1 if the mother is currently unemployed (ref cat: not in the labor force).	0.030<br>(0.171)	0.026<br>(0.160)
Labor Market Controls			
Unemployment – males	The unemployment rate of males, at the month of the LSAC respondents' interview, in the local labor market region (SA4) for which the LSAC respondent resides.	4.938<br>(2.104)	4.950<br>(2.105)
Unemployment – females	The unemployment rate of females, at the month of the LSAC respondents' interview, in the local labor market region (SA4) for which the LSAC respondent resides.	5.307<br>(2.257)	5.308<br>(2.271)
Emp to pop ratio – males	The employment to population ratio of males, at the month of the LSAC respondents' interview, in the local labor market region (SA4) for which the LSAC respondent resides.	68.297<br>(5.938)	68.257<br>(6.032)
Emp to pop ratio – females	The employment to population ratio of females, at the month of the LSAC respondents' interview, in the local labor market region (SA4) for which the LSAC respondent resides.	54.180<br>(5.999)	54.156<br>(6.026)

Table S2. Creating the analysis sample for children.

	No. of Children	Observations
Baseline sample	4,983	13,616
Drop observations with missing data for:		
the expectations shock		424
parent-reported SDQ measures		279
control variables		125
Resulting sample	4,862	12,788
less observations where the child is not present in both waves 1 & 2	773	1,094
Final estimating sample	4,089	11,694
No. of boys	2,084	5,960
No. of girls	2,005	5,734

Notes: Baseline sample consists of all LSAC cohort K children but restricts the observations to waves 1, 2 and 4.

Table S3. Creating the analysis sample for mothers.

	No. of Mothers	Observations
Baseline sample	4,853	13,176
Drop observations with missing data for:		
the expectations shock		400
psychological distress K6		1,069
control variables		443
Resulting sample	4,401	11,264
less observations where the mother is not present in both waves 1 & 2	1,080	1,702
Final estimating sample	3,321	9,562

Notes: Baseline sample consists primary female guardians of the children in cohort K (130 kids' primary caregiver was a male), over waves 1, 2 and 4.

Table S4. Tests of parallel trends between treatment and control, children.

	Parent-rated			Teacher-rated		
	SDQ total	Internalizing	Externalizing	SDQ total	Internalizing	Externalizing
	(1)	(2)	(3)	(4)	(5)	(6)
**A: Expectations shock: top 25th percentile**						
Expectations shock	0.023	0.020	0.018	0.082	0.150**	0.006
	(0.032)	(0.036)	(0.033)	(0.054)	(0.062)	(0.052)
Constant	-0.258***	-0.072***	-0.332***	0.016	-0.020	0.040*
	(0.014)	(0.016)	(0.015)	(0.024)	(0.027)	(0.023)
R-Square	0.000	0.000	0.000	0.000	0.001	0.000
N	4089	4089	4089	2256	2258	2261
**B: Expectations shock: >average**						
Expectations shock	0.014	0.002	0.019	0.026	0.070	-0.014
	(0.026)	(0.029)	(0.027)	(0.044)	(0.050)	(0.042)
Constant	-0.259***	-0.069***	-0.336***	0.022	-0.019	0.047*
	(0.017)	(0.019)	(0.017)	(0.028)	(0.032)	(0.027)
R-Square	0.000	0.000	0.000	0.001	0.003	0.000
N	4089	4089	4089	2256	2258	2261

Notes: Estimated OLS coefficients presented and standard errors in parentheses. *, **, *** indicates significance at the 10%, 5 % and 1% levels respectively.

Table S5. Tests of parallel trends between treatment and control, mothers.

	K6 Score
**A: Expectations shock: top 25th percentile**	
Expectations shock	0.116***
	(0.042)
Constant	-0.287***
	(0.019)
R-square	0.002
N	3321
**B: Expectations shock: >average**	
Expectations shock	0.025
	(0.034)
Constant	-0.275***
	(0.022)
R-square	0.000
N	3321

Notes: Estimated OLS coefficients presented and standard errors in parentheses. *, **, *** indicates significance at the 10%, 5 % and 1% levels respectively.

Table S6. Complete results for the of unemployment expectations shocks (top 25th percentile) on parent-rated SDQ outcomes, boys.

	SDQ total (1)	Internalizing (2)	Externalizing (3)	SDQ total (4)	Internalizing (5)	Externalizing (6)
Expectations shock	0.082**	0.021	0.108***	0.069*	0.016	0.092**
	(0.040)	(0.040)	(0.040)	(0.041)	(0.042)	(0.041)
Post-crisis	-0.056	-0.010	-0.076	-0.044	0.002	-0.068
	(0.127)	(0.128)	(0.127)	(0.128)	(0.128)	(0.127)
DID	0.002	-0.030	0.027	-0.002	-0.034	0.024
	(0.071)	(0.072)	(0.071)	(0.072)	(0.072)	(0.072)
Child's age	0.027	0.039	0.009	0.025	0.038	0.007
	(0.030)	(0.030)	(0.030)	(0.030)	(0.030)	(0.030)
Number of children	-0.073***	-0.093***	-0.034**	-0.074***	-0.094***	-0.035**
	(0.014)	(0.014)	(0.014)	(0.014)	(0.014)	(0.014)
Lone-parent	0.211***	0.163***	0.188***	0.212***	0.164***	0.188***
	(0.042)	(0.042)	(0.041)	(0.042)	(0.042)	(0.041)
Blended family	0.167**	0.149**	0.133*	0.168**	0.149**	0.134*
	(0.073)	(0.073)	(0.073)	(0.073)	(0.073)	(0.073)
Year 12 (mother)	-0.121**	-0.101**	-0.101**	-0.120**	-0.099**	-0.101**
	(0.048)	(0.048)	(0.048)	(0.048)	(0.048)	(0.048)
Certificate (mother)	-0.097**	-0.054	-0.103**	-0.094**	-0.052	-0.100**
	(0.041)	(0.041)	(0.040)	(0.041)	(0.041)	(0.040)
Diploma (mother)	-0.269***	-0.173***	-0.268***	-0.267***	-0.172***	-0.265***
	(0.053)	(0.054)	(0.053)	(0.054)	(0.054)	(0.053)
Bachelor or above (mother)	-0.260***	-0.112***	-0.303***	-0.257***	-0.110**	-0.300***
	(0.043)	(0.043)	(0.042)	(0.043)	(0.043)	(0.042)
Employed (mother)	-0.156***	-0.199***	-0.075**	-0.156***	-0.198***	-0.076**
	(0.031)	(0.031)	(0.031)	(0.031)	(0.031)	(0.031)
Unemployed (mother)	-0.022	-0.092	0.042	-0.025	-0.093	0.038
	(0.079)	(0.079)	(0.078)	(0.079)	(0.079)	(0.078)
Change in household	0.102**	0.062	0.105**	0.101**	0.061	0.103**
	(0.041)	(0.042)	(0.041)	(0.041)	(0.042)	(0.041)
Moved home	-0.036	-0.057	-0.008	-0.035	-0.056	-0.008
	(0.041)	(0.041)	(0.041)	(0.041)	(0.041)	(0.041)
Ln equiv.household weekly income	-0.115***	-0.146***	-0.056*	-0.117***	-0.148***	-0.058*
	(0.031)	(0.031)	(0.031)	(0.031)	(0.031)	(0.031)
Imputation flag	-0.008	-0.003	-0.010	-0.005	0.001	-0.008
	(0.070)	(0.071)	(0.070)	(0.070)	(0.071)	(0.070)
Missing income	-0.744***	-0.896***	-0.401*	-0.761***	-0.910***	-0.416*
	(0.217)	(0.218)	(0.216)	(0.217)	(0.219)	(0.216)
Home: Mortgage	0.077*	0.050	0.076*	0.076*	0.050	0.075*
	(0.041)	(0.041)	(0.041)	(0.041)	(0.041)	(0.041)
Home: Renting	0.235***	0.197***	0.196***	0.233***	0.197***	0.194***
	(0.050)	(0.050)	(0.050)	(0.050)	(0.050)	(0.050)
Home: Other	0.096	0.098	0.065	0.095	0.099	0.064
	(0.083)	(0.083)	(0.082)	(0.083)	(0.083)	(0.082)
SEIFA	-0.039***	-0.035***	-0.030***	-0.041***	-0.036***	-0.033***
	(0.006)	(0.006)	(0.006)	(0.006)	(0.006)	(0.006)
Inner regional	0.008	-0.012	0.022	0.022	-0.003	0.036
	(0.034)	(0.035)	(0.034)	(0.035)	(0.036)	(0.035)
Outer regional	-0.011	-0.020	-0.000	-0.010	-0.014	-0.004
	(0.045)	(0.046)	(0.045)	(0.046)	(0.046)	(0.046)
Remote	-0.086	0.038	-0.161	-0.066	0.043	-0.135
	(0.108)	(0.108)	(0.107)	(0.109)	(0.109)	(0.108)
Wave 1 missing	-0.468**	-0.813***	-0.051	-0.485**	-0.831***	-0.063
	(0.217)	(0.218)	(0.216)	(0.217)	(0.218)	(0.216)
Unemployment – males	-	-	-	0.004	0.009	-0.001
				(0.008)	(0.008)	(0.008)

Unemployment – females	-	-	-	0.001<br>(0.007)	-0.007<br>(0.007)	0.007<br>(0.007)
Emp to pop – males	-	-	-	0.008*<br>(0.004)	0.008**<br>(0.004)	0.005<br>(0.004)
Emp to pop – females	-	-	-	-0.003<br>(0.004)	-0.005<br>(0.004)	0.000<br>(0.004)
Constant	1.093***<br>(0.286)	1.195***<br>(0.288)	0.688**<br>(0.285)	0.735**<br>(0.357)	0.966***<br>(0.359)	0.332<br>(0.355)
R-Square	0.083	0.057	0.076	0.084	0.058	0.077
N	5960	5960	5960	5960	5960	5960

Notes: Estimated OLS coefficients presented and standard errors in parentheses. *, **, *** indicates significance at the 10%, 5 % and 1% levels respectively.

Table S7. Complete results for the effects of unemployment expectations shocks (top 25th percentile) on parent-rated SDQ outcomes, girls.

	SDQ total (1)	Internalizing (2)	Externalizing (3)	SDQ total (4)	Internalizing (5)	Externalizing (6)
Expectations shock	-0.026	-0.014	-0.029	-0.042	-0.034	-0.035
	(0.037)	(0.039)	(0.036)	(0.038)	(0.040)	(0.037)
Post-crisis	0.034	0.194	-0.106	0.044	0.209*	-0.103
	(0.113)	(0.119)	(0.112)	(0.113)	(0.119)	(0.112)
DID	0.133**	0.091	0.128**	0.152**	0.108	0.143**
	(0.066)	(0.069)	(0.065)	(0.066)	(0.070)	(0.065)
Child's age	-0.008	-0.023	0.006	-0.010	-0.026	0.005
	(0.027)	(0.028)	(0.027)	(0.027)	(0.028)	(0.027)
Number of children	-0.034***	-0.037***	-0.021*	-0.033***	-0.036***	-0.021*
	(0.012)	(0.013)	(0.012)	(0.012)	(0.013)	(0.012)
Lone-parent	0.283***	0.265***	0.214***	0.282***	0.264***	0.213***
	(0.038)	(0.040)	(0.037)	(0.038)	(0.040)	(0.037)
Blended family	0.253***	0.237***	0.192***	0.255***	0.239***	0.193***
	(0.066)	(0.069)	(0.065)	(0.066)	(0.069)	(0.065)
Year 12 (mother)	-0.131***	-0.169***	-0.063	-0.132***	-0.168***	-0.064
	(0.043)	(0.046)	(0.043)	(0.043)	(0.046)	(0.043)
Certificate (mother)	-0.063*	-0.041	-0.063*	-0.066*	-0.043	-0.065*
	(0.036)	(0.038)	(0.035)	(0.036)	(0.038)	(0.035)
Diploma (mother)	-0.207***	-0.130**	-0.209***	-0.207***	-0.129**	-0.209***
	(0.048)	(0.051)	(0.048)	(0.048)	(0.051)	(0.048)
Bachelor or above (mother)	-0.315***	-0.196***	-0.318***	-0.316***	-0.196***	-0.320***
	(0.037)	(0.039)	(0.037)	(0.037)	(0.039)	(0.037)
Employed (mother)	-0.140***	-0.153***	-0.089***	-0.138***	-0.149***	-0.088***
	(0.028)	(0.030)	(0.028)	(0.028)	(0.030)	(0.028)
Unemployed (mother)	-0.047	0.014	-0.082	-0.044	0.017	-0.081
	(0.072)	(0.076)	(0.071)	(0.072)	(0.076)	(0.071)
Change in household	0.044	0.045	0.030	0.044	0.045	0.030
	(0.037)	(0.039)	(0.036)	(0.037)	(0.039)	(0.036)
Moved home	-0.021	0.029	-0.055	-0.020	0.030	-0.055
	(0.036)	(0.038)	(0.035)	(0.036)	(0.038)	(0.035)
Ln equiv.household weekly income	-0.105***	-0.119***	-0.062**	-0.104***	-0.119***	-0.061**
	(0.028)	(0.029)	(0.028)	(0.028)	(0.029)	(0.028)
Imputation flag	-0.004	0.034	-0.033	-0.004	0.032	-0.033
	(0.061)	(0.064)	(0.060)	(0.061)	(0.064)	(0.060)
Missing income	-0.689***	-0.779***	-0.414**	-0.681***	-0.773***	-0.407**
	(0.192)	(0.203)	(0.190)	(0.192)	(0.203)	(0.190)
Home: Mortgage	0.097***	0.074**	0.086**	0.095***	0.073*	0.085**
	(0.035)	(0.037)	(0.035)	(0.035)	(0.037)	(0.035)
Home: Renting	0.224***	0.128***	0.235***	0.220***	0.125***	0.232***
	(0.043)	(0.046)	(0.043)	(0.043)	(0.046)	(0.043)
Home: Other	0.137*	0.070	0.151*	0.137*	0.071	0.150*
	(0.079)	(0.084)	(0.078)	(0.079)	(0.084)	(0.078)
SEIFA	-0.023***	-0.023***	-0.016***	-0.025***	-0.025***	-0.017***
	(0.005)	(0.005)	(0.005)	(0.006)	(0.006)	(0.005)
Inner regional	-0.065**	-0.065**	-0.046	-0.065**	-0.061*	-0.049
	(0.030)	(0.032)	(0.030)	(0.031)	(0.033)	(0.031)
Outer regional	-0.101**	-0.124***	-0.053	-0.099**	-0.117***	-0.055
	(0.042)	(0.044)	(0.041)	(0.042)	(0.044)	(0.042)
Remote	-0.271**	-0.359***	-0.120	-0.236**	-0.314***	-0.104
	(0.112)	(0.118)	(0.110)	(0.112)	(0.119)	(0.111)
Wave 1 missing	-0.429**	-0.766***	-0.030	-0.421**	-0.762***	-0.021
	(0.194)	(0.205)	(0.192)	(0.194)	(0.205)	(0.192)
Unemployment – males				-0.022***	-0.022***	-0.016**
				(0.007)	(0.008)	(0.007)

Unemployment – females				0.002	0.002	0.002
				(0.006)	(0.007)	(0.006)
Emp to pop – males				-0.003	0.001	-0.006*
				(0.003)	(0.004)	(0.003)
Emp to pop – females				-0.001	-0.004	0.002
				(0.003)	(0.004)	(0.003)
Constant	0.811***	1.163***	0.288	1.205***	1.447***	0.655**
	(0.255)	(0.270)	(0.253)	(0.322)	(0.341)	(0.319)
R-Square	0.108	0.062	0.113	0.110	0.063	0.113
N	5734	5734	5734	5734	5734	5734

Notes: Estimated OLS coefficients presented and standard errors in parentheses. *, **, *** indicates significance at the 10%, 5 % and 1% levels respectively.

Table S8. Complete results for the effects of unemployment expectations shocks on mother's psychological distress (Kessler 6).

	Model excluding local labor market controls		Model including local labor market controls	
	(1)	(2)	(3)	(4)
Expectations shock (25th percentile)	0.061**		0.077**	
	(0.031)		(0.032)	
Expectations shock (>average)		-0.022		-0.018
		(0.025)		(0.026)
Post-crisis	0.104***	0.092***	0.098***	0.087***
	(0.028)	(0.032)	(0.029)	(0.032)
DID	-0.069	-0.004	-0.074	-0.006
	(0.055)	(0.044)	(0.056)	(0.044)
Mother's age	0.001	0.001	0.001	0.001
	(0.002)	(0.002)	(0.002)	(0.002)
Number of children	-0.013	-0.012	-0.013	-0.012
	(0.011)	(0.011)	(0.011)	(0.011)
Lone-parent	0.291***	0.290***	0.289***	0.288***
	(0.033)	(0.033)	(0.033)	(0.033)
Blended family	0.036	0.037	0.035	0.036
	(0.062)	(0.062)	(0.062)	(0.062)
Change in household	0.049	0.049	0.049	0.049
	(0.032)	(0.032)	(0.032)	(0.032)
Moved home	0.065**	0.067**	0.064**	0.066**
	(0.032)	(0.032)	(0.032)	(0.032)
Ln equiv.household weekly income	-0.183***	-0.182***	-0.182***	-0.180***
	(0.024)	(0.024)	(0.024)	(0.024)
Imputation flag	0.005	0.002	0.004	0.002
	(0.054)	(0.054)	(0.054)	(0.054)
Missing income	-1.177***	-1.163***	-1.167***	-1.155***
	(0.169)	(0.169)	(0.169)	(0.169)
Home: Mortgage	0.077**	0.077**	0.076**	0.076**
	(0.030)	(0.030)	(0.030)	(0.030)
Home: Renting	0.154***	0.156***	0.154***	0.155***
	(0.039)	(0.039)	(0.039)	(0.039)
Home: Other	0.152**	0.154**	0.151**	0.152**
	(0.067)	(0.067)	(0.067)	(0.067)
SEIFA	-0.016***	-0.016***	-0.014***	-0.015***
	(0.004)	(0.004)	(0.005)	(0.005)
Inner regional	-0.138***	-0.135***	-0.148***	-0.141***
	(0.026)	(0.026)	(0.027)	(0.027)
Outer regional	-0.056	-0.043	-0.062*	-0.048
	(0.035)	(0.035)	(0.036)	(0.035)
Remote	-0.188**	-0.139*	-0.210**	-0.148*
	(0.086)	(0.084)	(0.087)	(0.085)
Wave 1 missing	-0.918***	-0.907***	-0.912***	-0.900***
	(0.161)	(0.161)	(0.161)	(0.161)
Year 12	-0.042	-0.040	-0.043	-0.041
	(0.037)	(0.037)	(0.037)	(0.037)
Certificate	0.048	0.049	0.047	0.049
	(0.031)	(0.031)	(0.031)	(0.031)
Diploma	0.027	0.028	0.026	0.028
	(0.041)	(0.041)	(0.041)	(0.041)
Bachelor or above	0.061*	0.062*	0.061*	0.061*
	(0.032)	(0.032)	(0.032)	(0.032)
Employed	-0.171***	-0.171***	-0.173***	-0.173***
	(0.024)	(0.024)	(0.024)	(0.024)
Unemployed	0.062	0.063	0.060	0.061
	(0.064)	(0.064)	(0.064)	(0.064)

Unemployment – males	-	-	0.004	0.001
			(0.006)	(0.006)
Unemployment – females	-	-	0.003	0.004
			(0.006)	(0.006)
Emp to pop – males	-	-	-0.005*	-0.005
			(0.003)	(0.003)
Emp to pop – females	-	-	0.004	0.004
			(0.003)	(0.003)
Constant	1.116***	1.123***	1.207***	1.168***
	(0.186)	(0.186)	(0.249)	(0.248)
R-Square	0.061	0.061	0.062	0.061
N	9562	9562	9562	9562

Notes: Estimated OLS coefficients presented and standard errors in parentheses. *, **, *** indicates significance at the 10%, 5 % and 1% levels respectively.
